# *Cannabis sativa*-mediated biosynthesis of copper-zinc-selenium nanocomposites: phytochemical profiling, antioxidant properties, and molecular docking insights

**DOI:** 10.1186/s42238-025-00320-9

**Published:** 2025-10-23

**Authors:** Ayesha Siddiqa, Rahmatullah Qureshi, Saima Kalsoom, Imtiaz Ahmad Khan, Zia ur Rehman Mashwani, Noor-Ul-Ain Zafar, Muhammad Bashir Farhan, Luis Edwardo Palomo Martínez, Ajaz Ahmad, Amir Ali

**Affiliations:** 1 Department of Botany, Pir Mehr Ali Shah Arid Agriculture University, Rawalpindi, Pakistan; 2 Department of Chemistry, Pir Mehr Ali Shah Arid Agriculture University, Rawalpindi, Pakistan; 3 Department of Pharmacy, University of Padova, Padova, Italy; 4 Department of Veterinary Pathology, Pir Mehr Ali Shah Arid Agriculture University, Rawalpindi, Pakistan; 5 Departamento de Ingeniería Bioquímica, Microencapsulación: Interacciones, Estructura, Función. Av. Wilfrido Massieu 399, Nueva Industrial Vallejo, Ciudad de México, CDMX, 07738 Mexico; 6 Department of Clinical Pharmacy, College of Pharmacy, King Saud University, Riyadh, 11451 Saudi Arabia

**Keywords:** Green synthesis, *Cannabis sativa*, Trimetallic nanocomposite, Copper-zinc-selenium, Antioxidant activity, Molecular docking

## Abstract

**Background:**

Nanotechnology is an emerging field widely applied across various disciplines, including medicine, to develop cost-effective and innovative therapies by delivering therapeutic compounds to targeted sites. The integration of multiple metals can yield synergistic multifunctional properties.

**Aims:**

This study focuses on the green synthesis of copper-zinc-selenium (Cu-Zn-Se) nanocomposite, which is a straightforward and reliable method compared to chemical, physical, and mechanical techniques. *Cannabis sativa*, a dioecious plant from the Cannabinaceae family, has garnered significant attention due to its pharmacological properties and global cultivation.

**Methods:**

In this research, Phytochemical analysis of extracts was carried out using Gas chromatography-mass spectrometry analysis (GC-MS). After that, a trimetallic copper-zinc-selenium (Cu-Zn-Se) nanocomposite was successfully synthesized using the floral biomass extract of *Cannabis sativa* and characterized by using UV-Vis spectral analysis, Scanning Electron Microscopy (SEM), and FTIR spectroscopy. Further, the potential applications of the synthesized trimetallic nanoparticles were evaluated by assessing their antioxidant activities through DPPH Free Radical Scavenging Activity, Hydrogen Peroxide Scavenging Assay, Ferric Reducing Power Assay, and Phosphomolybdate Antioxidant Activity. In addition, Molecular docking studies were used to investigate the drug target interactions for antioxidant behaviour.

**Results:**

The UV-Vis spectrum displayed overlapping bands at 230, 290, and 327 nm, confirming the successful synthesis of the nanocomposite. FTIR analysis revealed peaks corresponding to various functional groups, notably a band at 1621.37 cm − 1 indicating C = O carbonyl stretching from amides, and a band at 1427.91 cm − 1 associated with C = C stretching (in-ring) from aromatic structures. SEM imaging showed spherical particles with an average size of 40 to 60 nm. A dose-dependent increase in antioxidant activity for the Cu-Zn-Se nanocomposite, which surpassed that of the plant extract in all assays studied and was comparable to the standard ascorbic acid. Molecular docking studies supported the experimental findings by showing that the Cu-Zn-Se nanocomposite binds stably to the antioxidant target protein, suggesting enhanced antioxidant activity.

**Conclusion:**

This study is among the first to report the green synthesis of a *Cannabis sativa*-mediated Cu-Zn-Se trimetallic nanocomposite, highlighting its strong antioxidant potential and interaction pathways at the molecular level. These findings contribute novel insights into sustainable nanomaterial development and underscore the biomedical promise of phytogenic trimetallic nanocomposites as potent antioxidant agent.

## Graphical Abstract

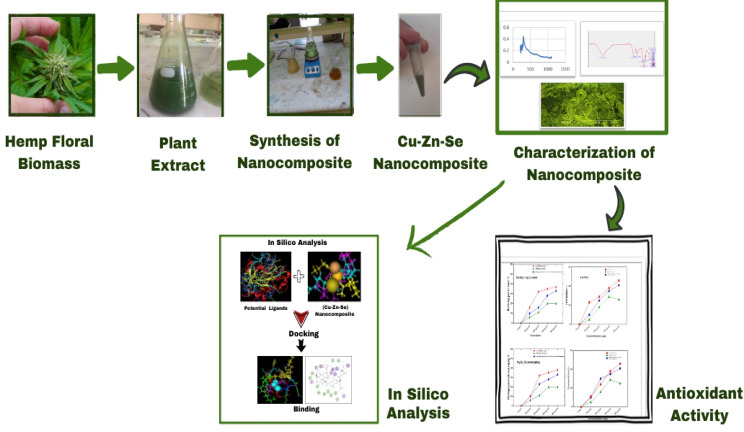

## Introduction

Nanotechnology refers to the division of science and engineering devoted to designing, creating, and application of structures, machines, and systems by the manipulation of atoms and molecules at the nanoscale, i.e. having one or more dimensions of the order of 100 nanometres (100 millionths of a millimeter) or less (Hornyak et al. 2018). In recent decades, these nanomaterials have contributed significantly to the exponential growth of nanoscience, green chemistry, and nano-biotechnology (Omran 2020); (Periakaruppan et al. 2023a, b, c). Nanotechnology is an emerging field of science popularly used in various disciplines including medicine for providing cost-effective and novel therapies by delivering therapeutic compounds to target site (Iftikhar et al. 2024; Siddiqa et al. 2024). However, nanomaterials frequently deviate considerably from their macroscale counterparts regarding their biological, physical, and chemical characteristics despite possessing similar chemical compositions (Thiruvengadathan et al. 2013). Various researchers used metal nanoparticles because of their broad application in medicine, biology, as well as in physics (Jain et al. 2008). Metal NPs are classified as monometallic (single metal), bimetallic (two metals), or trimetallic NPs based on the number of metals/metal oxides involved.

In comparison to mono- and bimetallic nanomaterials, trimetallic NPs have recently gained interest in the present era due to their exceptional features, strong qualities, and enhanced physiochemical properties; for example, their physical, chemical, optical, and magnetic properties due to the combined synergistic effects of their metal (Mahmood et al. 2022); (Joseph et al. 2025). The enormous natural availability and inexpensive price of copper make Cu NPs particularly alluring. Cu-based nanocatalysts have several uses in nanotechnology due to their special traits and attributes, such as catalysing organic transformations, electrocatalysis, and photocatalysis. Cu NPs have been shown to have amazing antioxidant properties in many of the studies performed. Additionally, Zinc (Rehman et al. 2024) and selenium nanoparticles possess significant antioxidant activity as reported by various studies (Ge et al. 2022).

Therefore, on the basis of the above-mentioned facts, the present study aimed to synthesized Cu-Zn-Se nanocomposite from floral biomass of hemp for evaluation of their structural and chemical characteristic through different characterization technique and to assess their antioxidant potential.

The development of methods for synthesizing NPs varying sizes and shapes has become an essential aspect of nanotechnology. Because of industrialization, urbanization, and population increase, harmful and undesired compounds are being emitted into the atmosphere, causing it to deteriorate. Physical and chemical methods of nanoparticle synthesis have been proven exorbitant and possibly precarious to the environment (Iftikhar et al. 2024). Synthesis of nanoparticles by green method, mainly plant extracts, is under exploitation. NPs synthesized from plants are more stable than those synthesized chemically. Reduction of metal precursors by biogenic means to corresponding NPs has many advantages including environmentally sustainable (Siddiqa et al. 2024), harmonious, pollutants free, cost-effective and amenable to large-scale production (Rana et al. 2020); (Sakthivel et al. 2025).

**Fig. 1:**
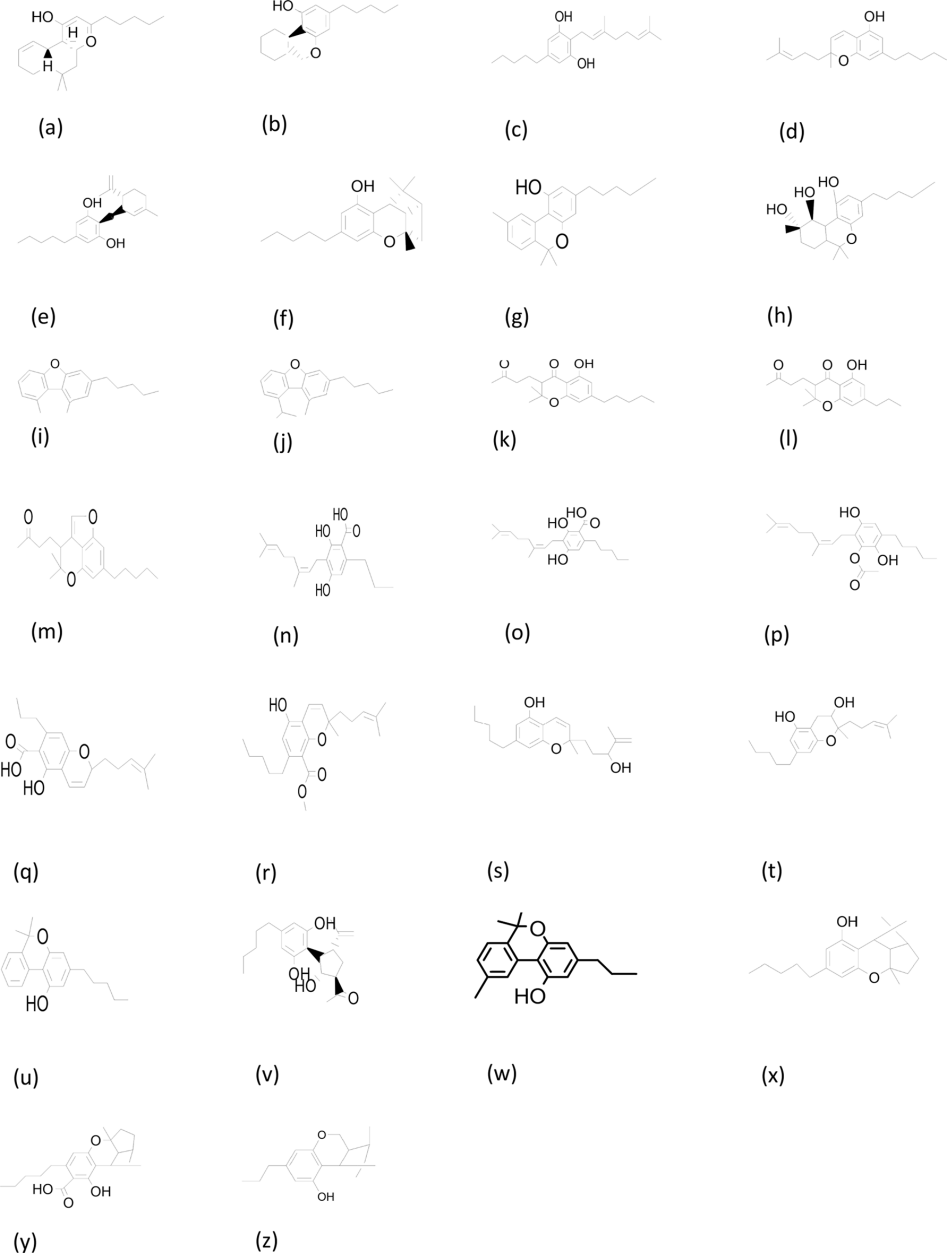
Phytocannabinoids reported in extract of *Cannabis sativa. *(**a**) ∆^9^-Tetra hydrocannabinol, (**b**) ∆^8^-Tetra hydrocannabinol, (**c**) Cannabigerol, (**d**) Cannabichromene, (**e**) Cannabidiol, (**f**) Cannabicyclol, (**g**) Cannabinol, (**h**) Cannabitriol, (**i**) Dehydrocannabifuran (**j**) Cannabifuran, (**k**) Cannabichromanon, (**l**) Bisnor cannabichromanon (**m**) Cannabicoumaronone (**n**) Cannabigerovarinic acid, (**o**) Y-Eudesmyl cannabigerolate, (**p**) a-Cadinl cannabigerolate, (**q**) Cannabichrome verinic, (**r**) 4-Acetoxycannabichrome, (**s**) 3-Hydroxy cannabichromene, (**t**) 7-Hydroxy cannabichromene, (**u**) Cannabinol, (**v**) Cannabimovone, (**w**) Cannabivarin, (**x**) Cannabicyclol, (**y**) Cannabicyclolic acid, (**z**) Cannabicyclovarin

The reduction of metal ions as well as capping of the synthesized nanomaterials (NMs) are known to be caused by primary and secondary metabolites, which include amino acids, enzymes, polysaccharides, phenolic and flavonoid compounds, terpenoids, alkaloids and cannabinoids. The influence of phytocompounds aids in the ability to manipulate the composition, size, and shape of NMs to control their function (Ahmed et al. 2022) (. The current study involved the use of *Cannabis sativa* floral biomass for synthesis of Cu-Zn-Se nanocomposite. *Cannabis sativa* is a dioecious plant belong to family cannabinaceae, it has gained special attention due to its pharmacological properties and cultivated worldwide. Medicinal properties of plant are attributed to secondary metabolites such as terpenoids, sugars, alkaloids, stilbenoids, quinones and the specific compounds of this plant, namely cannabinoids (Sulak 2021). This is a unique and specific group of compounds found in cannabis plant and responsible for management of variety of diseases. These phytochemicals possess significant antioxidant potential. They are produced by glandular trichomes mostly present on female inflorescence. Until now more than 100 cannabinoids have been isolated and identified (De et al., 2012). Among these, the most reported compounds have been shown in Fig. 1. Two major compounds Cannabidiol (CBD) and tetrahydrocannabinol (THC) have been extensively explored for their role in treatment of various diseases. These cannabinoids exert their ameliorating effects through interaction with endocannabinoids system (Lalsare 2022).

To date, only a limited number of researchers have reported on the antioxidant potential of copper, zinc and selenium NPs using various parts of hemp. Further, although only a few reports have been published on synthesizing Cu-Zn-Se NC using biological approaches, antioxidant potential of plant mediated Cu-Zn-Se NC has not reported yet. However, few studies are available on antioxidant potential of individual Cu, Zn and Se nanoparticles only using DPPH assay. However, those results were also not comprehensively reported (Gunti et al. 2019; Mahendra et al. 2020; Rehman et al. 2022). Hence, an attempt was made to synthesize biogenic NCs combining three types of NPs, Cu, Zn, and Se. and compare the antioxidant potential of NCs to the respective plant extract. To the best of our knowledge, this remains the first report on comparative study on hemp-based Cu-Zn-Se nanocomposite.

## Materials and methods

### Chemical and reagents

The chemicals and reagents used in this study include ethanol (C₂H₅OH), zinc nitrate (Zn(NO₃)₂), copper nitrate (Cu(NO₃)₂), sodium selenite (Na₂SeO₃), sodium hydroxide (NaOH), DPPH (1,1-diphenyl-2-picrylhydrazyl, C₁₈H₁₂N₅O₆), hydrogen peroxide (H₂O₂), potassium ferricyanide (K₃[Fe(CN)₆]), phosphate buffer, trichloroacetic acid (CCl₃COOH), ferric chloride (FeCl₃), sulfuric acid (H₂SO₄), ammonium molybdate ((NH₄)₆Mo₇O₂₄·4 H₂O), sodium phosphate (Na₃PO₄), ascorbic acid (C₆H₈O₆).

### Sample collection and Preparation of plant extract

Plant Sample was provided by Plant Taxonomy Lab, Department of Botany, PMAS Arid agriculture university, Rawalpindi, Pakistan. To prepare plant extract, Inflorescences was allowed to air dry before 10 g is weighed, added to 100 ml of ethanol, and submerged in an ultrasonic bath for 30 min to enhance the extraction efficiency by disrupting plant cell walls, thereby facilitating the release of bioactive compounds into the solvent. Subsequently, the mixture was filtered through three rounds of Whatman No. 1 filter paper, to obtain clear filtrate. The liquids obtained after filtration was evaporated under reduced pressure using a low temperature, and the dry extract was obtained and then stored in an airtight container at 4 °C until further use in subsequent experiments. (Giselle et al. 2023).

#### GC-MS analysis

Gas chromatography–mass spectrometry (GC-MS) analysis was performed at the Centralized Resource Laboratory, University of Peshawar, Pakistan, using a PerkinElmer Clarus 500 system with helium as the carrier gas. The GC setup included a DB-5ms capillary column (30 m × 0.25 mm × 0.25 μm), with a 1 µL injection volume delivered via an autosampler at a split ratio of 20:1. The inlet temperature was maintained at 280 °C, with a total run time of 36 min. The oven temperature was initially held at 70 °C for 3 min, then ramped at 10 °C/min to a final temperature of 300 °C. MS analysis was conducted in scan mode with a mass range of 150–650 m/z, using a solvent delay of 2 min. The MS source temperature was set at 250 °C, while the quadrupole temperature was maintained at 200 °C. Compound identification was performed using the MassHunter/NIST 2017 spectral library.

Cannabinoids present in hemp were identified by comparing their retention times and peak areas. The mass spectra obtained from GC-MS analysis were interpreted using the National Institute of Standards and Technology (NIST) database (https://www.nist.gov/m), with compound identification carried out through ChemStation software and the NIST.L compound library. To confirm the identified compounds, their retention times and molecular weights were cross-referenced with existing literature. Additionally, molecular weights, chemical formulas, and compound identifiers were retrieved from the PubChem database (https://pubchem.ncbi.nlm.nih.gov/) for further validation.

### Synthesis of the Cu-Zn-Se nanocomposite

**Fig. 2:**
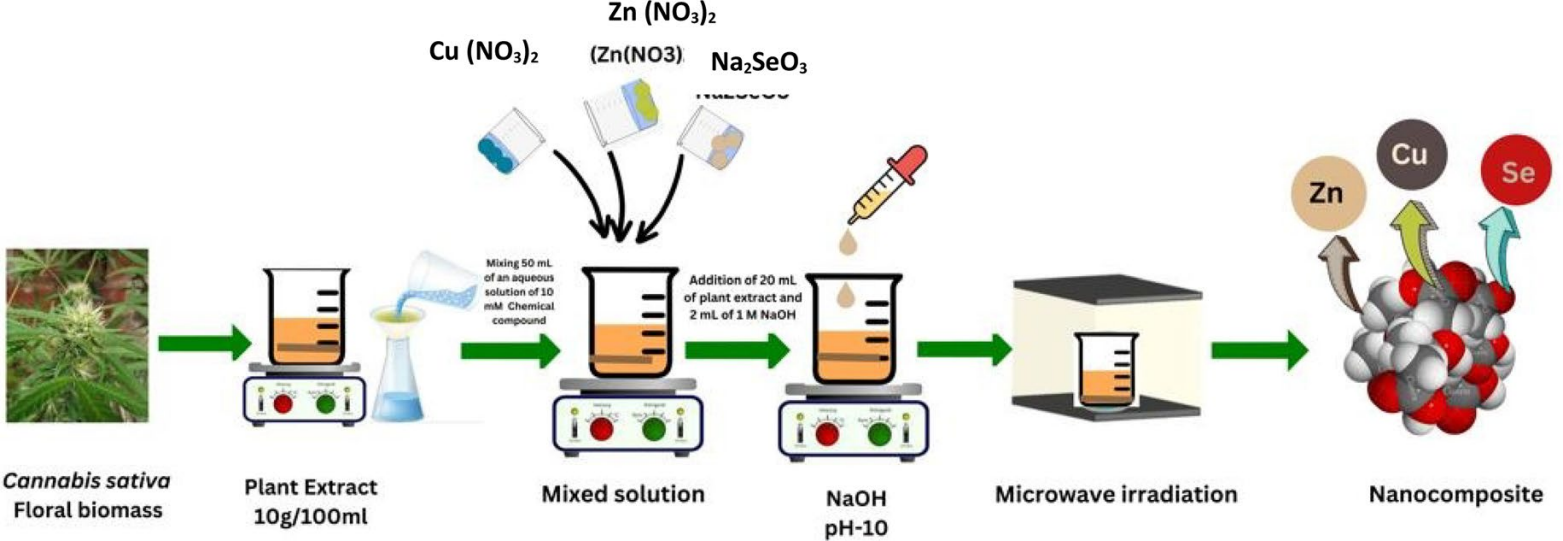
Biosynthesis of Cu-Zn-Se nanocomposite using hemp floral biomass

The synthesis of the Cu-Zn-Se nanocomposite involves combining 50 ml of a 10 mM aqueous solution of Zn (NO_3_)_2_, 50 ml of a 10 mM aqueous solution of Cu (NO_3_)_2_, and 50 ml of a 10 mM aqueous solution of Na_2_SeO_3_. Subsequently, the mixture was continuous stirred at a temperature of 80–90 °C, followed by the addition of 20 ml of leaf extract and 2 ml of 1 M NaOH to adjust the alkalinity of the reaction medium, which enhances the nucleation and growth of nanoparticles by facilitating the reduction of metal ions and promoting the formation of metal hydroxide intermediates. The pH of the reaction mixture was maintained in the alkaline range of approximately 9–10 during the stirring process to ensure optimal conditions for nanocomposite synthesis. Stirring persists until a change in the solution’s color was observed (Fig. 2). The resulting colloidal solution was put to centrifugation, and the pellet was obtained and dried using a vacuum drier at 70–80 °C for 24 h (Mohammadi-Aloucheh et al. 2018); (Periakaruppan et al. 2023a, b, c).

### Characterization

#### UV–Vis spectrum analysis

UV-Vis spectroscopy analysis between 200 and 700 nm was used to study the bio-reduction of metal salts to their elemental form by using UV-Vis spectrophotometer (Beckman DU640 UV- spectrophotometer). For sample preparation, 1 mg/ml of the synthesized Cu-Zn-Se nanocomposite was dispersed in deionized water and sonicated for 10–15 min to achieve a uniform suspension and prevent particle agglomeration. The resulting colloidal solution was then used for spectral analysis. The absorption spectrum was recorded.

#### Fourier transform-infrared (FT-IR) analysis

Using an FT-IR spectrophotometer (NICOLET 6700, Thermo, Waltham, MA, USA) with a resolution of 0.15 cm-1. For sample preparation, a small quantity of the dried nanocomposite powder was finely ground and mixed thoroughly with potassium bromide (KBr) at a 1:100 ratio (sample: KBr), then pressed into a translucent pellet using a hydraulic press. A spectral scan analysis was conducted across wave numbers spanning from 400 to 4000 cm-1 to ascertain the primary bioactive compound in *C. sativa*. This compound is presumed to play a role in the creation of a nanocomposite and investigate the functional groups of the material.

#### Scanning electron microscopy (SEM)

The morphology of the produced nanocomposite was examined using a Scanning Electron Microscope (SEM) (SEM, JSM5910 JEOL, Tokyo, Japan) at an accelerating voltage of 15 kV to investigate its surface structure, shape, size, and overall topography.

### Antioxidant analysis

Green synthesized Cu-Zn-Se nanocomposite was subjected for the screening of antioxidant potential. Readings were taken in three replicates for each sample. For characterization and estimation of antioxidant activity following antioxidant assays were conducted for Cu-Zn-Se nanocomposite synthesized from floral biomass of *Cannabis sativa* in comparison to reference standard test sample of Ascorbic acid.

#### DPPH free radical scavenging activity

DPPH (1,1-Diphenyl-2-picrylydrazyl) is a stable free radical which gives deep violet colour and an absorption band at 517 nm in methanolic solution. When mixed with any compound of antioxidative potential for 30 min DPPH loses its deep violet colour (Alam et al. 2013). This assay was performed following the method of Khoshnamvand et al. (2019) with slight modifications. Various concentrations of each extract ranging from 50 to 400 µg/ml were allowed to react with 0.1 mM DPPH methanolic solution and percentage inhibition of DPPH radical was calculated using following formula:

$${\rm{\% }}\,{\rm{age }}\,{\rm{inhibition }}\,{\rm{of }}\,{\rm{DPPH }}\,{\rm{radical = }}{{{{\rm{A}}_{\rm{C}}}{\rm{ - }}{{\rm{A}}_{\rm{T}}}} \over {{{\rm{A}}_{\rm{C}}}}} \times 100$$

Where A_C_ is the absorbance of control and A_T_ is the absorbance of test sample.

#### Hydrogen peroxide scavenging assay

In Hydrogen peroxide Scavenging Assay, method of (Rajoka et al. 2020) was followed with slight modification by incubating a mixture of 0.8 ml (40 mM) H_2_O_2_ soln. with 2 ml soln. of varying concentrations (10–200 g/ml) of extracts and standard ascorbic acid reference sample for 30 min and then % age inhibition of H_2_O_2_ at 230 nm was calculated by using following formula:

$${\rm{\% }}\,{\rm{age }}\,{\rm{inhibition }}\,{\rm{of }}\,{{\rm{H}}_{\rm{2}}}{{\rm{O}}_{\rm{2}}}\,{\rm{radical }} = {{{\rm{H}}{{\rm{P}}_{\rm{C}}}{\rm{ - H}}{{\rm{P}}_{\rm{T}}}} \over {{\rm{H}}{{\rm{P}}_{\rm{C}}}}} \times 100$$

Where HP_C_ is absorbance of control and HP_T_ is absorbance of test sample.

#### Ferric reducing power assay

For this analysis, method used by (Keshari et al. 2020) was employed in which % age inhibition was evaluated for varying concentrations (50–400 µg/ml) of 2.5 ml sample solution and standard ascorbic acid reference sample in 1% potassium ferrocyanate in 0.2 M phosphate buffer (1:1) were mixed and incubated for 20 min at 65 ℃. The mixture is centrifuged at 3000 rpm for 10 min after adding 2.5 ml of 10% Trichloroacetic acid. After centrifugation 2.5 ml distilled water and 0.5 ml of 1% ferric chloride is added to the reaction mixture or to supernatant if formed. OD was taken at 700 nm and for calculating % age inhibition following formula was used:

$$\% \,{\rm{age }}\,{\rm{inhibition}}\,{\rm{ of}}\,{\rm{ Hydroxyl}}\,{\rm{ radical = }}{{{F_C} - {F_T}} \over {{F_C}}} \times 100$$

Whereas F_C_ is absorbance of control and F_T_ is absorbance of test sample.

#### Phosphomolybdate antioxidant activity

For phosphomolybdate antioxidant activity analysis methodology of Moonmun et al. (2017) was used with slight modifications for evaluation of 0.3 ml of test samples and standard reference ascorbic acid sample of varying concentrations (50–400 µg/ml) in 3 ml phosphomolybdate reagent which was prepared by mixing equal volumes of 0.6 M sulphuric acid, 4 mM ammonium molybdate and 28 mM sodium phosphate. Reaction mixture is incubated for 90 min in dark at 95 ℃. OD was measured at 765 nm and % age inhibition was calculated by using following formula:

$${\rm{\% }}\,{\rm{ age}}\,{\rm{ inhibition }}\,{\rm{of}}\,{\rm{ phosphomolybdate }} = {{{\rm{P}}{{\rm{M}}_{\rm{C}}}{\rm{ - P}}{{\rm{M}}_{\rm{T}}}} \over {{\rm{P}}{{\rm{M}}_{\rm{C}}}}} \times 100$$

Whereas PM_C_ is absorbance of control and PM_T_ is absorbance of test sample.

### In-silico studies

Molecular docking studies were used to investigate the drug target interactions for.

antioxidant behavior. Human antioxidant target protein PDB Id: 3MNG was used for docking findings. Nanoparticle structure was constructed by using ChemDraw software and geometry was optimized by energy minimizing tool of MOE. Cu-Zn-Se composite with plant metabolites were drawn by using software and saved in database. The docking study was carried out by the protein loaded in Molecular Operating Environment (MOE) and the errors of the protein were corrected by the structure preparation processes in MOE. After the corrections, the ligand molecules were removed and the polar hydrogen atoms were added to the isolated target with their standard geometry. Energy minimization was performed for both ligands and target upto 0.05 kcal/mol and the lowest energy conformer was selected as the global minimal for modeling study. Docking simulation was performed and ten conformations were generated for Cu-Zn-Se nanocomposite and reference drug.

ascrobic acid.

## Results and discussion

### GC-MS analysis

**Fig. 3:**
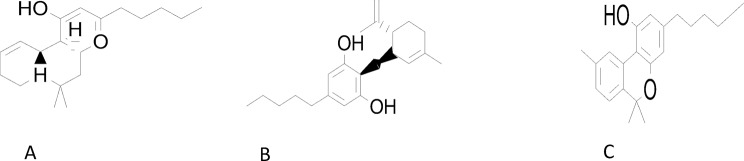
Phytometabolites found in *Cannabis sativa* L. (**A**) Δ9-Tetrahydrocannabinol, (**B**) Cannabidiol, (**C**) Cannabinol

The gas chromatography-mass spectrometry (GC-MS) analysis of the ethanol extract of Cannabis sativa revealed a diverse and complex phytochemical profile, prominently featuring three major cannabinoids: cannabidiol (CBD), cannabinol (CBN), and Δ9-tetrahydrocannabinol (Δ9-THC). These cannabinoids are among the most pharmacologically active secondary metabolites found in cannabis and contribute significantly to its therapeutic potential. The GC-MS chromatogram showed that Δ9-THC was detected with a retention time of 17.68 min and a corresponding mass-to-charge ratio (m/z) of 299, consistent with previously reported findings (Huestis 2002). Cannabidiol (CBD) was identified at a retention time of 16.6 min, marked by a distinct m/z peak at 231. Additionally, cannabinol (CBN), known as a primary oxidative degradation product of THC, appeared at a retention time of 18.4 min with a notable m/z 271 peak, indicating potential exposure to oxidative conditions during extraction or storage. These observations align with earlier reports by Park et al. (2022), who highlighted floral tissues as the richest in cannabinoid content, and by Isahq et al. (2015) and Basas-Jaumandreu et al. (2020), who similarly documented the prevalence of THC, CBD, and CBN in significant concentrations in Cannabis sativa samples. Furthermore, a recent study by Safir et al. (2025) also confirmed the detection of cannabinoids using ethanol extracts analyzed via GC-MS, supporting the reliability of this extraction and detection method. In addition to cannabinoids, the GC-MS spectrum displayed characteristic m/z peaks at 55 and 91, which are indicative of monoterpenes such as limonene and myrcene—two compounds known for their aromatic properties and synergistic interactions with cannabinoids. The presence of these terpenes reinforces the entourage effect theory, which suggests that cannabinoids and terpenes work together to produce enhanced therapeutic outcomes. Overall, the detailed identification of cannabinoids and associated terpenes underscores the phytochemical richness of Cannabis sativa. These findings not only validate the extract’s bioactivity but also emphasize the need for precise profiling of bioactive constituents in cannabis-based therapeutic formulations (See Fig. 3).

### Characterization of silver nanoparticles

**Fig. 4:**
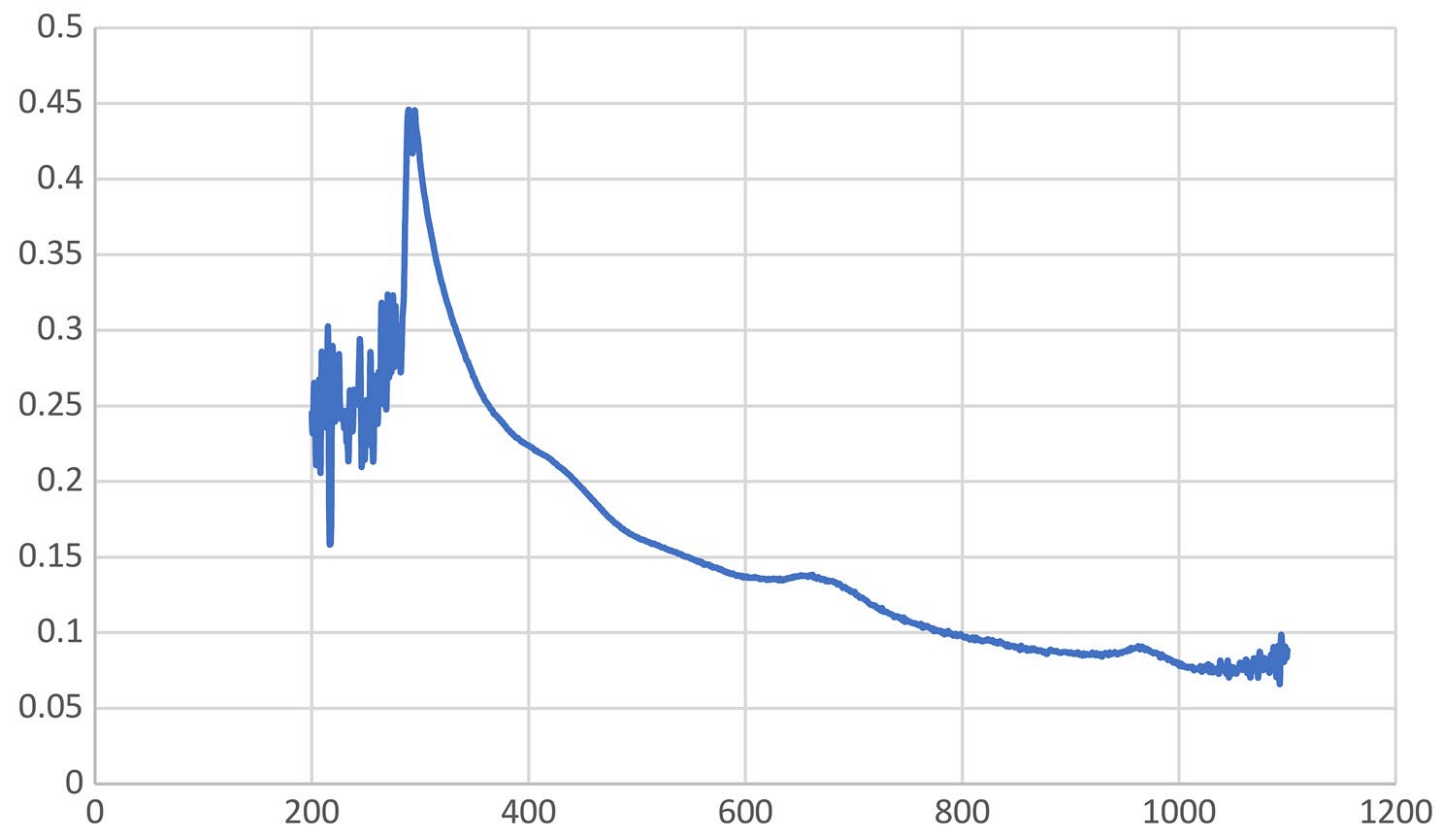
UV spectrum of green synthesized Cu-Zn-Se nanocomposite

An efficient technique for keeping track of the development of a reaction or studying the surface plasmon resonance (SPR) of metal nanoparticles is ultraviolet–visible (UV–Vis) spectrophotometry (Ganeshan et al. 2024). It is a method by which light absorption passes through an experimental sample. Color change shows the synthesis of nanoparticles (Fig. 2). Both the ultraviolet and visible spectrums of light are energetic and capable of raising the energy level of electrons. In this study, the wavelength range of 0–1200 nm was used to measure the absorbance. ultraviolet–visible spectrum of the Cu-Zn-Se nanocomposite was illustrated in Fig. 4, it confirmed the presence of the three nanometals without convention bands but in the range of peaks related to each metal. In particular, the bands at 230, 290, 327 confirmed the presence of CuONPs, ZnONPs, and SeNPs, respectively, but without a specific recording because the bands overlap (Cao et al. 2021; Hashem and Salem 2022; Kaur et al. 2022; Surendra et al. 2021). In this manner, the obtained results prospectively confirm the biosynthesis and integration of the trimetallic structure.

**Fig. 5:**
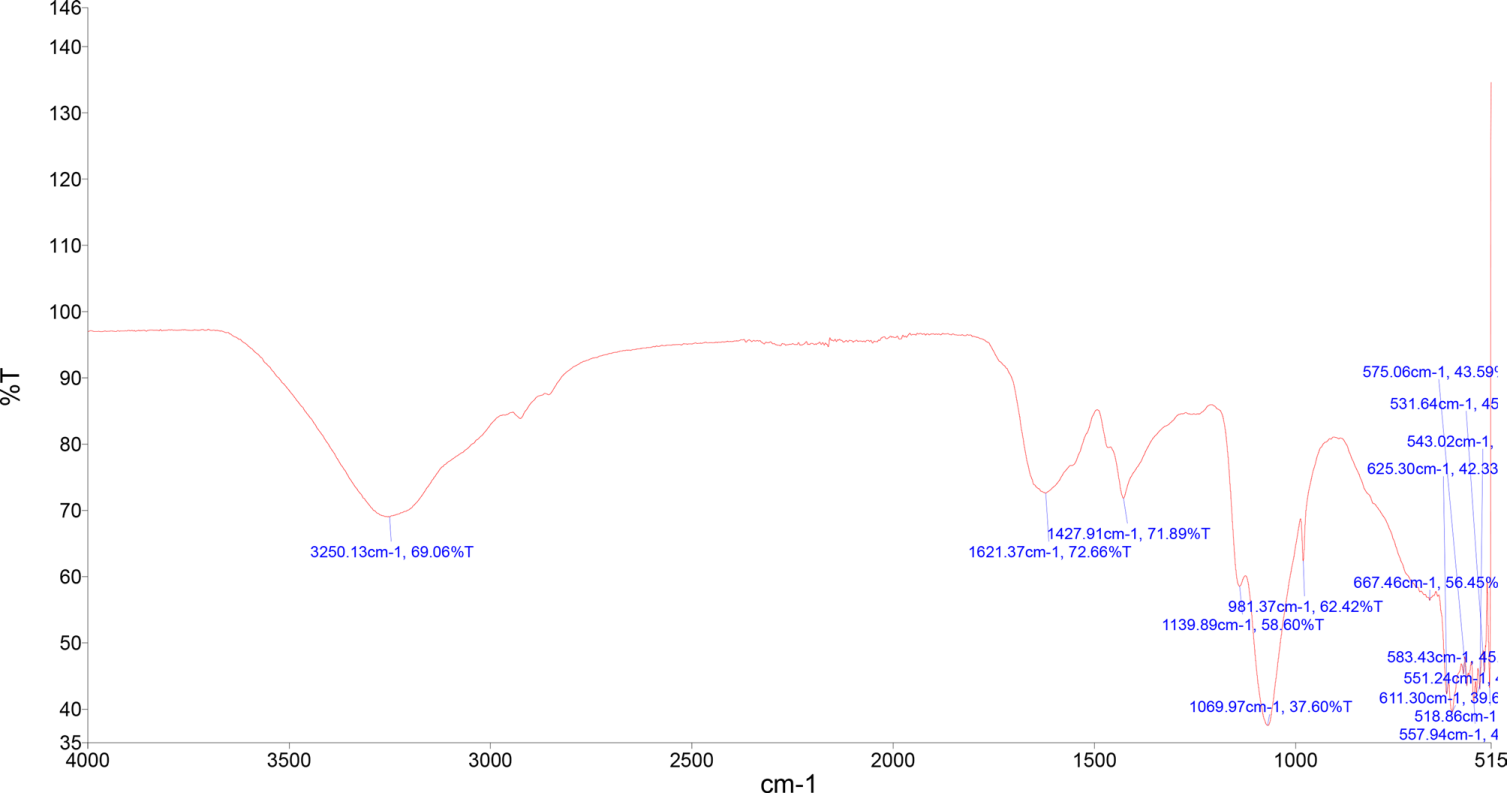
FTIR of green synthesized Cu-Zn-Se nanocomposite

FT-IR spectroscopic analysis was performed to determine the major functional groups present on the surface of phytogenic nanocomposite and in the *C. sativa* floral biomass extract and their possible involvement in the synthesis and stabilization of nanocomposite. The analysis of the obtained spectra reflects the presence of strong band at 3250.13 cm^− 1^, it is associated with –OH stretching vibration from alcohols (Mittal et al. 2014; Singh et al. 2018). Between 1700 and 1500 cm^− 1^, a large and strong bands were observed, such as band at 1621.37 cm^− 1^ correspond to C = O carbonyl stretching from amides and band at 1427.91 cm^− 1^ associated with C = C stretching (in-ring) from aromatic structures. Similar behavior was reported by Singh et al. (2018) for the green synthesis of gold and silver nanoparticles from *Cannabis sativa* (industrial hemp) and this band was assigned to C = O carbonyl stretching and C = C stretching. Characteristic absorption at 1139.89 cm^− 1^ represents the C-O stretching due to the presence of tertiary alcohol, while 1069.97 cm^− 1^ represents the C-O stretching due to the presence of primary alcohol (Suneetha 2018) (Fig. 5).

**Fig. 6:**
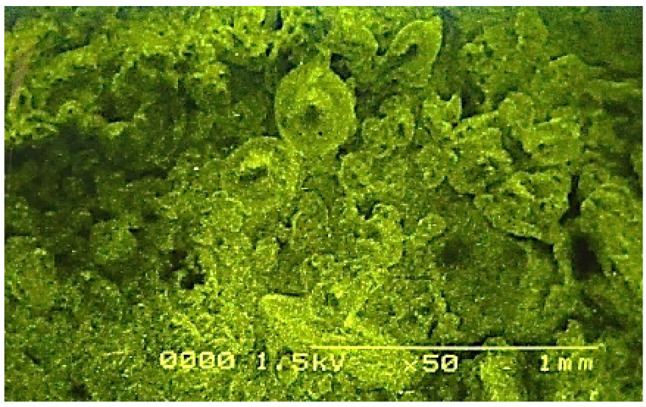
Scanning Electron Micrograph (SEM) of green synthesized

The presence of Cu, Zn and Se was confirmed by SEM analysis, which is shown in the figure given below (Fig. 6). The SEM micrographs of the trimetallic Cu-Zn-Se nanocomposite biosynthesised using the floral biomass extract of *Canabis sativa* showed spherical shapes with a size ranging from 50 to 77 nm with average size of about 60 nm (Figs. 6). A similar trimetallic nanocomposite synthesized using fungal extract was also characterized through topographical analysis, which confirmed the micromorphology and particle size of the nanoparticles (Hashem et al. 2023).

### Antioxidant activity analysis

#### DPPH free radical scavenging activity analysis

**Fig. 7:**
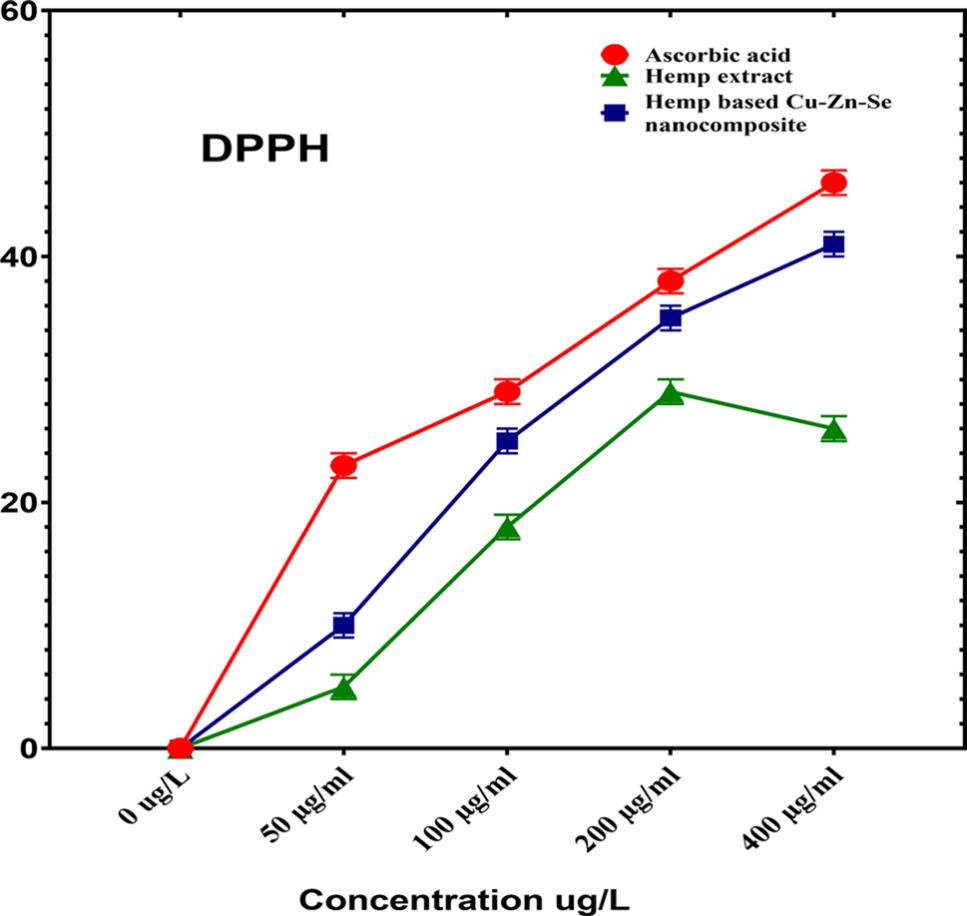
DPPH free radical scavenging activity of Cu-Zn-Se nanocomposite, hemp extract and ascorbic acid at different concentrations

To determine the antioxidant activity of the Cu-Zn-Se nanocomposite a DPPH assay was performed in which ascorbic acid was used as a positive control (Fig. 7). In the DPPH scavenging activity assay, Cu-Zn-Se nanocomposite and plant extract were tested in triplicates across 50, 100, 200, and 400 µg·ml^–1^ concentration vs. ascorbic acid. For concentrations of 50, 100 and 200 µg·ml–1, inhibitions were 10 ± 0.02%, 25 ± 0.01% and 35 ± 0.01% for Cu-Zn-Se nanocomposite, and 5 ± 0.01%, 18 ± 0.01% and 29 ± 0.01% for plant extract, Ascorbic acid demonstrated pronounced antioxidant activity, with inhibitions of 23 ± 0.01%, 29 ± 0.01% and 38 ± 0.02% at these concentrations. At 400 µg·ml^–1^, Cu-Zn-Se nanocomposite and plant extract exhibited inhibitions of 41 ± 0.02% and 26 ± 0.01%, respectively, with ascorbic acid at a significant 46 ± 0.01% (Figs. 7) highlighting ascorbic acid’s superior antioxidant capacity. Furthermore, at higher concentrations (400 µg/ml), the Cu-Zn-Se nanocomposite exhibited remarkably high antioxidant activity compared to lower concentrations, same trend was observed for ascorbic acid. But plant extract showed highest antioxidant activity at 200 µg/ml concentration. The synthesis, characterisation, and antioxidant and antibacterial activity of copper nanoparticles were reported in a study using C. vitiginea leaf extract. The antioxidant activity of the synthesised copper nanoparticles was recorded to be 21% (Wu et al. 2020). Consistent with our findings, results reported by Nguyen et al. in which maximum antioxidant activity for CuONPs, ZnONPs and CuO/ZnO nanocomposite was observed at highest concentration (400 µg/ml) (Nguyen et al. 2023). Moreover, the antioxidant activities of CuO nanoparticles synthesized based on *Annona muricata* leaf extract, demonstrated robust DPPH inhibition with IC50 values of 131.59 µg/ml (Awan et al. 2023). Another study reported the DPPH scavenging activity of selenium nanoparticles in dose dependent manner, with maximum potential at highest dose (Kong et al. 2014); (Periakaruppan et al. 2025). In another study, trimetallic gold/silver/copper nanoparticles were synthesised, and their antioxidant activity was evaluated using the DPPH assay. The nanoparticles exhibited strong antioxidant activity, with higher scavenging activity than individual gold, silver, and copper nanoparticles (Kunwar et al. 2023); (Periakaruppan et al. 2025).

#### Hydrogen peroxide scavenging activity analysis

**Fig. 8:**
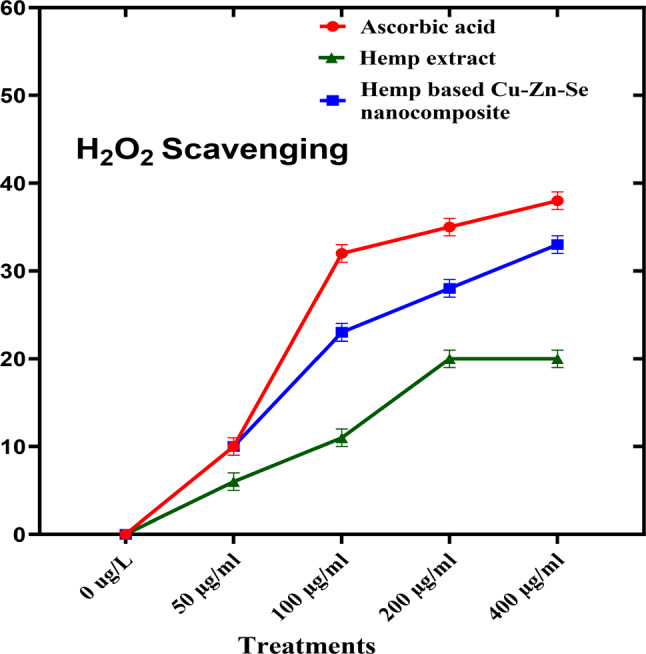
Hydrogen Peroxide Scavenging activity of Cu-Zn-Se nanocomposite, hemp extract and ascorbic acid at different concentrations

Hydrogen peroxide scavenging activity analysis was performed to determine the antioxidant activity of the Cu-Zn-Se nanocomposite in which ascorbic acid was used as a positive control (Fig. 8). In this assay, Cu-Zn-Se nanocomposite and plant extract were tested in triplicates across 50, 100, 200, and 400 µg·ml^–1^ concentration vs. ascorbic acid. For concentrations of 50, 100 and 200 µg·ml–1, inhibitions were 10 ± 0.02%, 23 ± 0.01% and 28 ± 0.01% for Cu-Zn-Se nanocomposite, and 6 ± 0.01%, 11 ± 0.01% and 20 ± 0.01% for plant extract, Ascorbic acid demonstrated pronounced antioxidant activity, with inhibitions of 10 ± 0.01%, 32 ± 0.01% and 34 ± 0.02% at these concentrations. At 400 µg·ml^–1^, Cu-Zn-Se nanocomposite and plant extract exhibited inhibitions of 33 ± 0.02% and 20 ± 0.01%, respectively, with ascorbic acid at a significant 38 ± 0.01% (Fig. 8) highlighting ascorbic acid’s superior antioxidant capacity. Furthermore, the Cu-Zn-Se nanocomposite exhibited remarkably high antioxidant activity at higher concentrations (400 µg/ml), compared to lower concentrations, same trend was observed for ascorbic acid. And antioxidant activity of Cu-Zn-Se nanocomposite was found comparable to standard ascorbic acid. But plant extract showed maximum antioxidant activity at 200 and 400 µg/ml concentration. The findings of current study are comparable to the previous reported by Aardra in which *Camellia sinensis*-mediated copper oxide nanoparticles showed a proportionate increase in antioxidant property with the increase in concentration compared to the standard (Aardra et al. 2023). Another study reported the hydrogen peroxide scavenging activity of selenium nanoparticles in dose dependent manner, with maximum potential at highest dose (Kong et al. 2014). Mahendran et al. reported the significant hydrogen peroxide scavenging activity of Zinc oxide (ZnO) nanoparticles synthesized by using aqueous extracts of *Aloe vera* (Mahendiran et al. 2017), these results are in strong coherence with current study.

#### Ferric reducing antioxidant power analysis

**Fig. 9:**
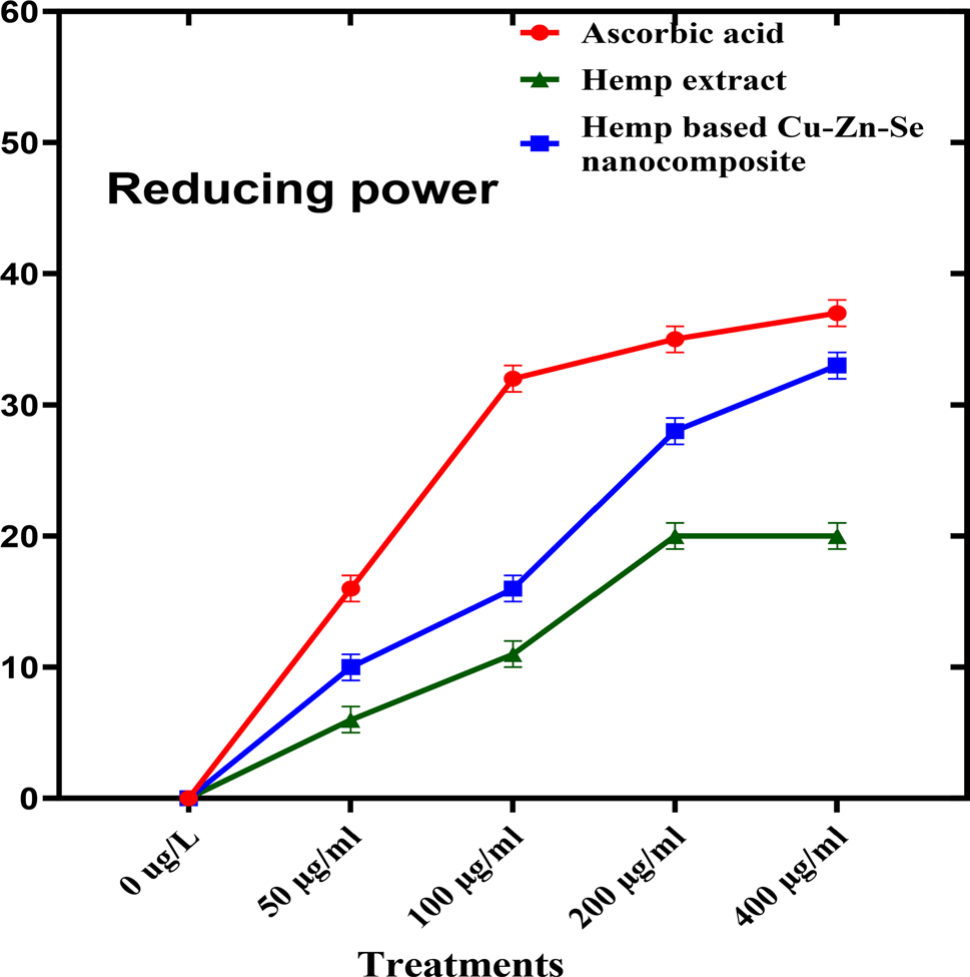
Ferric reducing antioxidant power analysis of Cu-Zn-Se nanocomposite, hemp extract and ascorbic acid at different concentrations

To determine the ferric reducing antioxidant power activity of the Cu-Zn-Se nanocomposite, ascorbic acid was used as a positive control. Cu-Zn-Se nanocomposite and plant extract were tested in triplicates across 50, 100, 200, and 400 µg·ml^–1^ concentration vs. ascorbic acid. For concentrations of 50, 100 and 200 µg·ml–1, inhibitions were 10 ± 0.02%, 16 ± 0.01% and 28 ± 0.01% for Cu-Zn-Se nanocomposite, and 6 ± 0.01%, 11 ± 0.01% and 20 ± 0.01% for plant extract, Ascorbic acid demonstrated pronounced antioxidant activity, with inhibitions of 16 ± 0.01%, 32 ± 0.01% and 35 ± 0.02% at these concentrations. At 400 µg·ml^–1^, Cu-Zn-Se nanocomposite and plant extract exhibited inhibitions of 33 ± 0.02% and 20 ± 0.01%, respectively, with ascorbic acid at a significant 37 ± 0.01% (Fig. 9) highlighting ascorbic acid’s superior antioxidant capacity. Furthermore, at higher concentrations (400 µg/ml), the Cu-Zn-Se nanocomposite exhibited remarkably high antioxidant activity compared to lower concentrations, same trend was observed for ascorbic acid. But plant extract showed highest antioxidant activity at 200 and 400 µg/ml concentration. Overall ferric reducing antioxidant power activity of Cu-Zn-Se nanocomposite was comparable to standard but it was higher than plant extract. The findings of current study supported by the results reported by Das et al. where the ferric reducing antioxidant power activity of CuO nanoparticles increased with increase in concentration (Das et al. 2013).

#### Phosphomolybdate antioxidant activity analysis

**Fig. 10:**
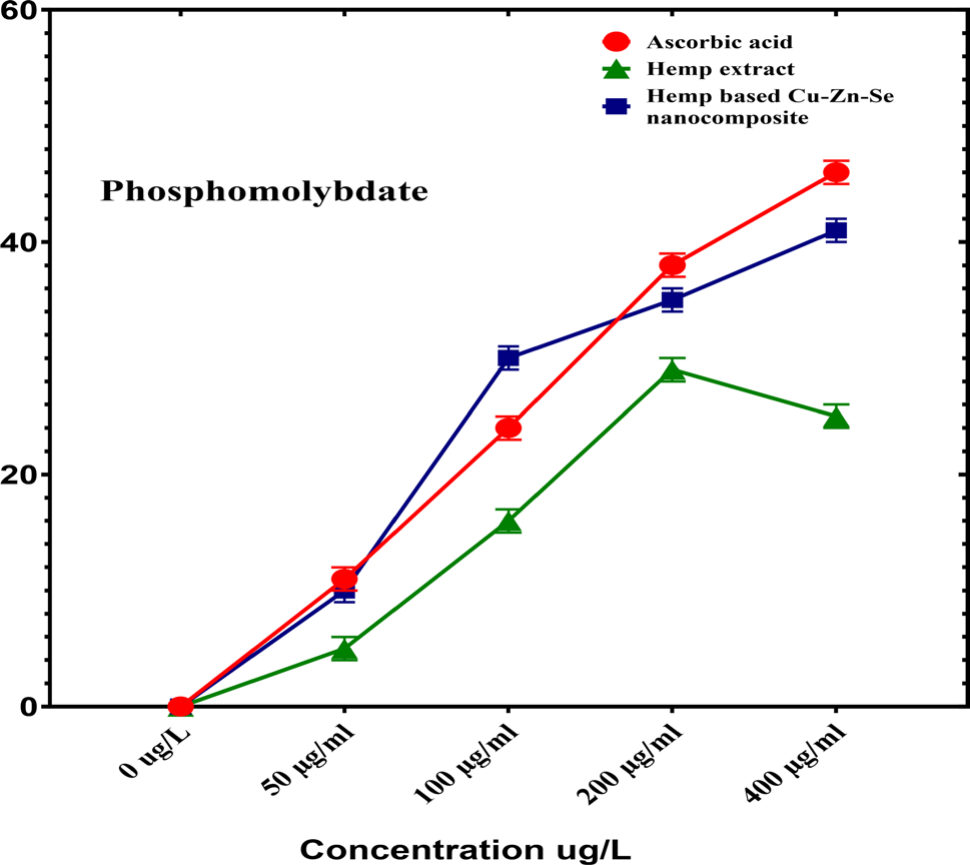
Phosphomolybdate activity analysis of Cu-Zn-Se nanocomposite, hemp extract and ascorbic acid at different concentrations

Phosphomolybdat activity analysis was performed to determine the antioxidant activity of the Cu-Zn-Se nanocomposite in which ascorbic acid was used as a positive control (Fig. 10). In this assay, Cu-Zn-Se nanocomposite and plant extract were tested in triplicates across 50, 100, 200, and 400 µg·ml^–1^ concentration vs. ascorbic acid. For concentrations of 50, 100 and 200 µg·ml–1, inhibitions were 10 ± 0.02%, 30 ± 0.01% and 35 ± 0.01% for Cu-Zn-Se nanocomposite, and 5 ± 0.01%, 17 ± 0.01% and 29 ± 0.01% for plant extract, Ascorbic acid demonstrated pronounced antioxidant activity, with inhibitions of 11 ± 0.01%, 24 ± 0.01% and 38 ± 0.02% at these concentrations. At 400 µg·ml^–1^, Cu-Zn-Se nanocomposite and plant extract exhibited inhibitions of 41 ± 0.02% and 25 ± 0.01%, respectively, with ascorbic acid at a significant 46 ± 0.01% (Figs. 10) highlighting ascorbic acid’s superior antioxidant capacity. Furthermore, the Cu-Zn-Se nanocomposite exhibited remarkably high antioxidant activity at higher concentrations (400 µg/ml), compared to lower concentrations, same trend was observed for ascorbic acid. And antioxidant activity of Cu-Zn-Se nanocomposite was found higher than plant extract but comparable to standard ascorbic acid. But plant extract showed maximum antioxidant activity at 200 µg/ml concentration. The green synthesis of nanoparticles has attracted considerable interest as an environmentally friendly and sustainable alternative to traditional chemical and physical methods. It utilizes biological resources like plants, bacteria, fungi, and algae, reducing the reliance on harmful chemicals, lowering energy requirements, and enhancing nanoparticle biocompatibility—features that make it especially valuable for biomedical and environmental uses (Kirubakaran et al. 2025a, b); (Kirubakaran et al. 2023). Findings of current study are comparable to previous reported by Ali et al. where green synthesized Se nanoparticles showed maximum phosphomolybdate antioxidant activity at higher concentration and it was higher than plant extract (Ali et al. 2024). The results of another study are in strong coherence with current findings, where phosphomolybdate antioxidant activity of copper, zinc nanoparticles and their composite were compared, Cu-Zn nanocomposite showed highest activity as compare to individual nanoparticles (Dubey et al. 2023) (Periakaruppan et al. 2023a, b, c).

### In-silico studies

**Fig. 11:**
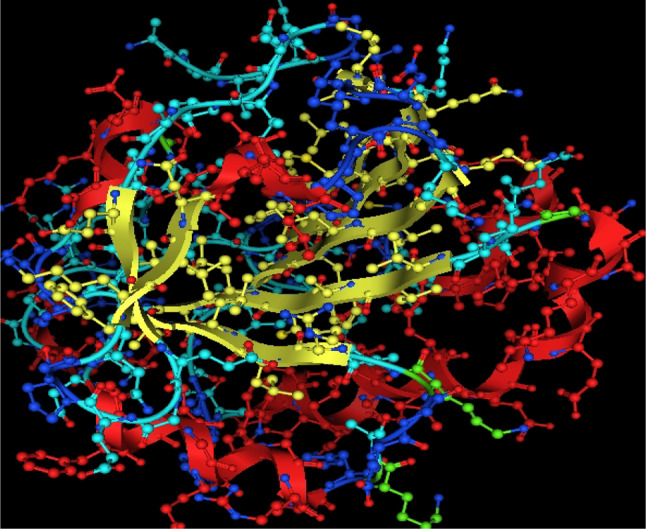
3D view of antioxidant target PDB id: 3MNg

**Fig. 12:**
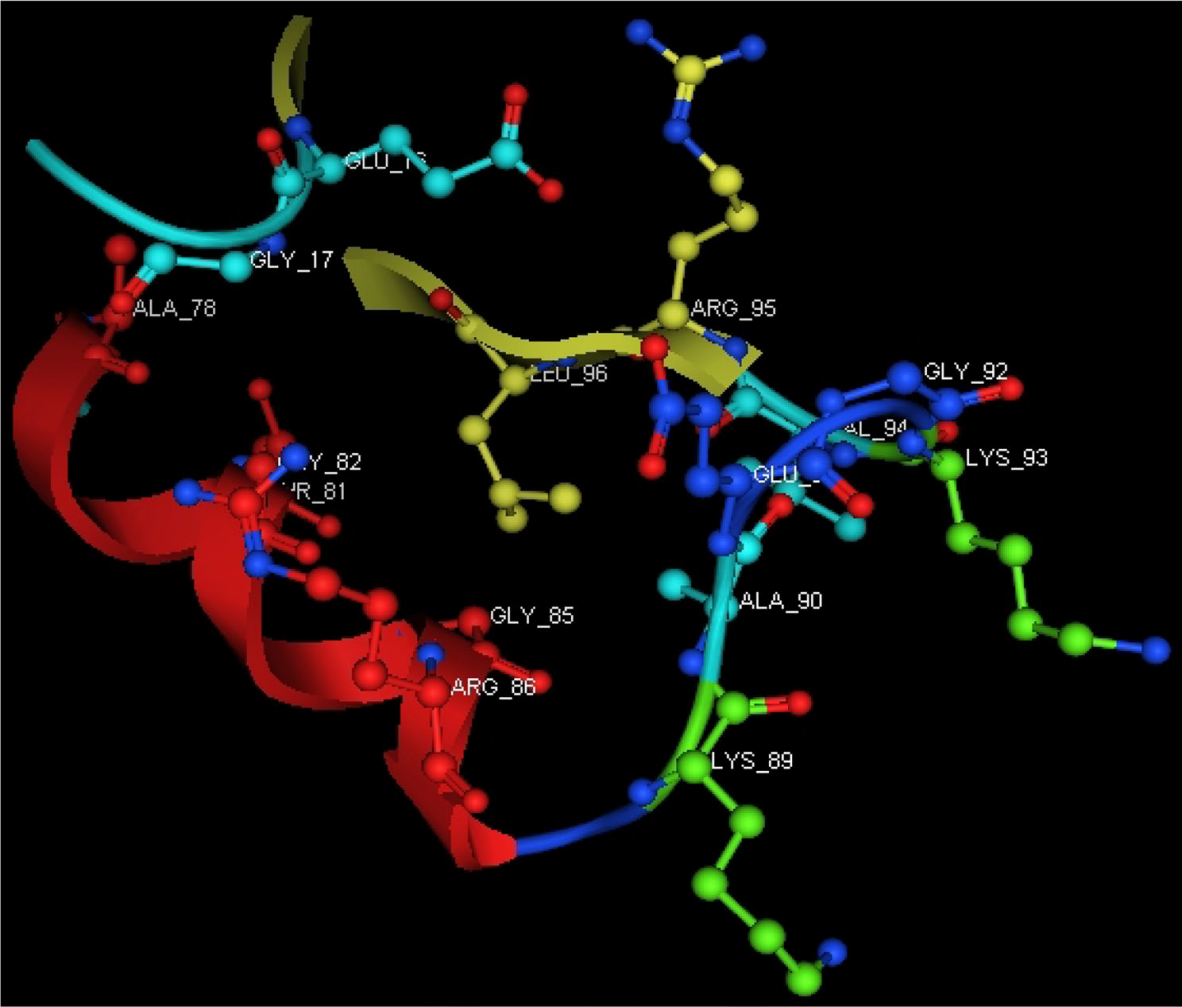
Active site of antioxidant protein

**Fig. 13:**
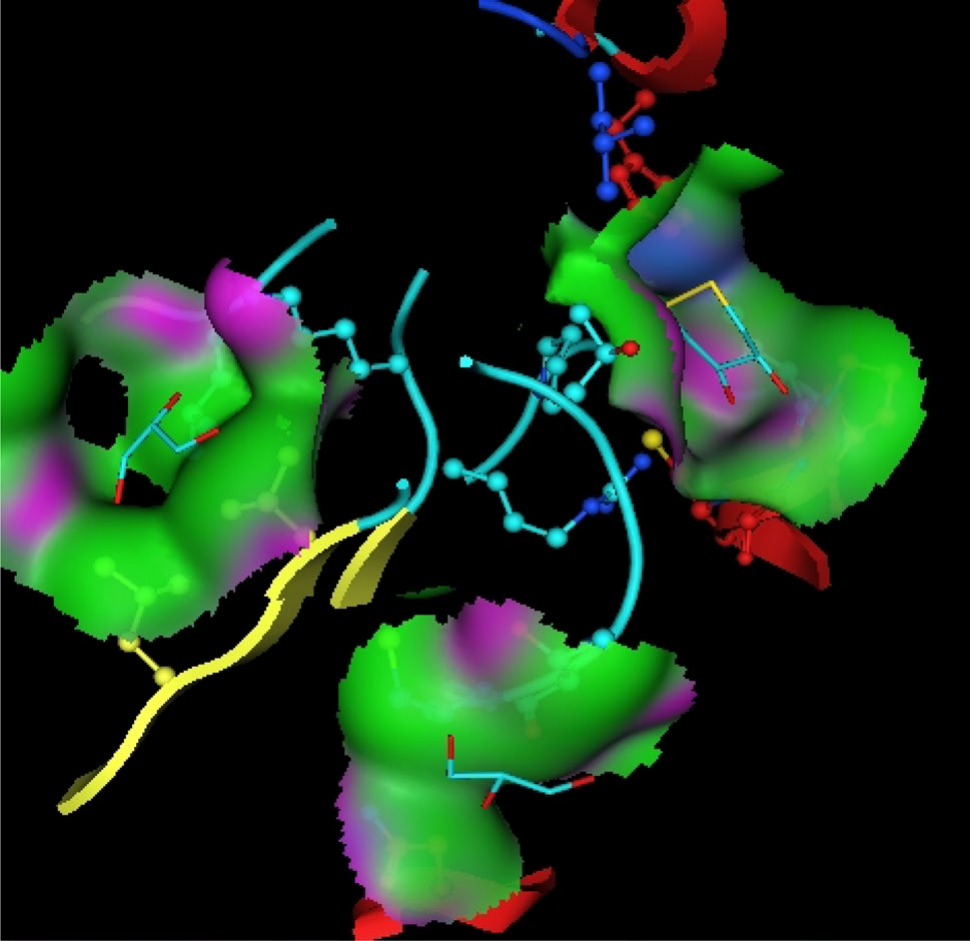
Surface map of binding pocket

**Fig. 14:**
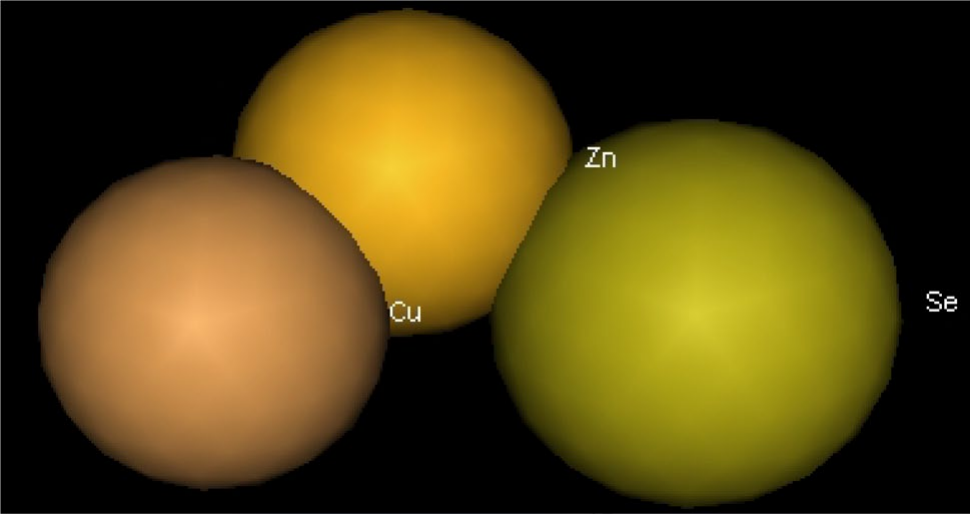
Cu-Zn-Se nanocomposite 2D view

**Fig. 15:**
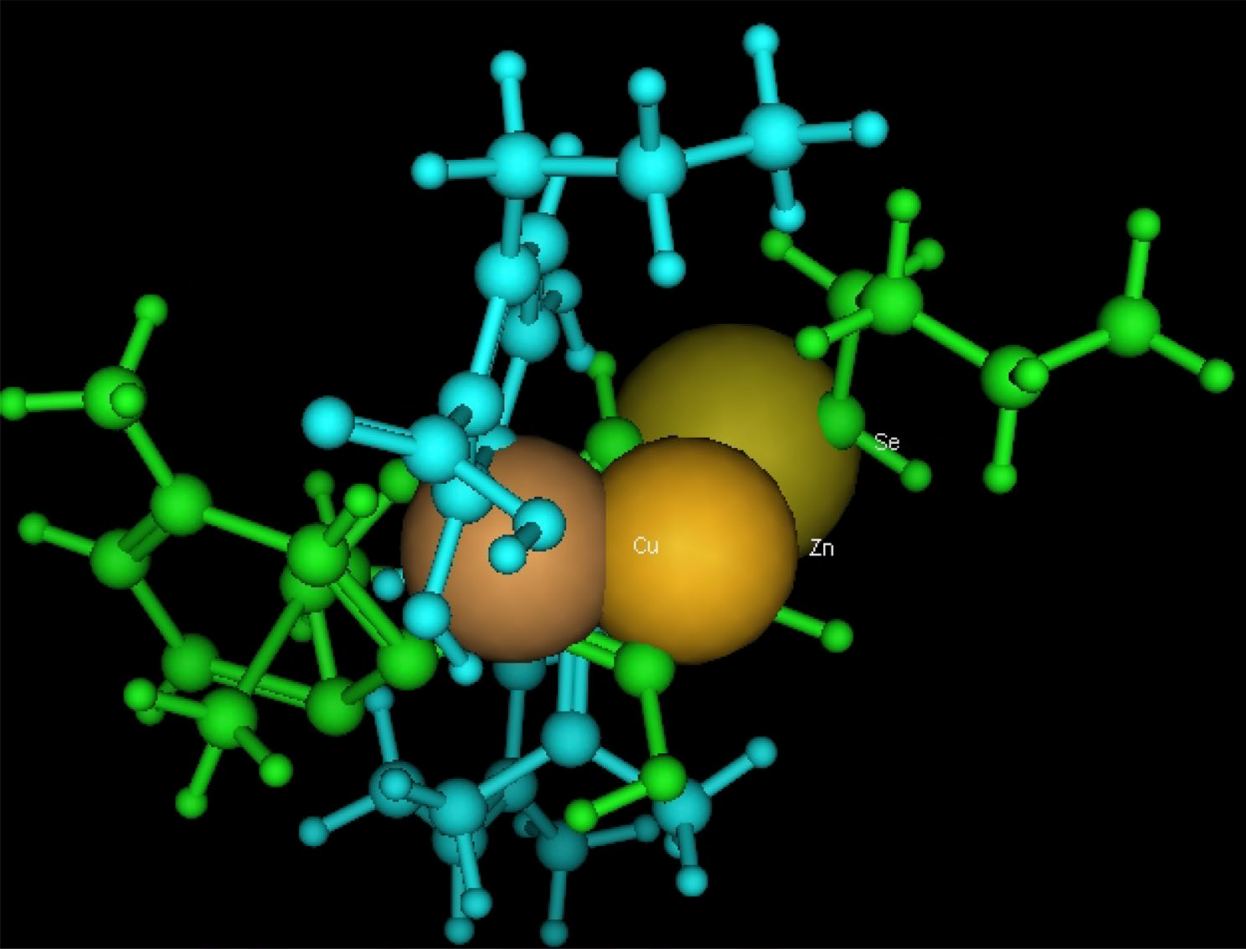
Copper-Zinc-Selenium (Cu-Zn-Se) nanocomposite with active plant metabolites

**Fig. 16:**
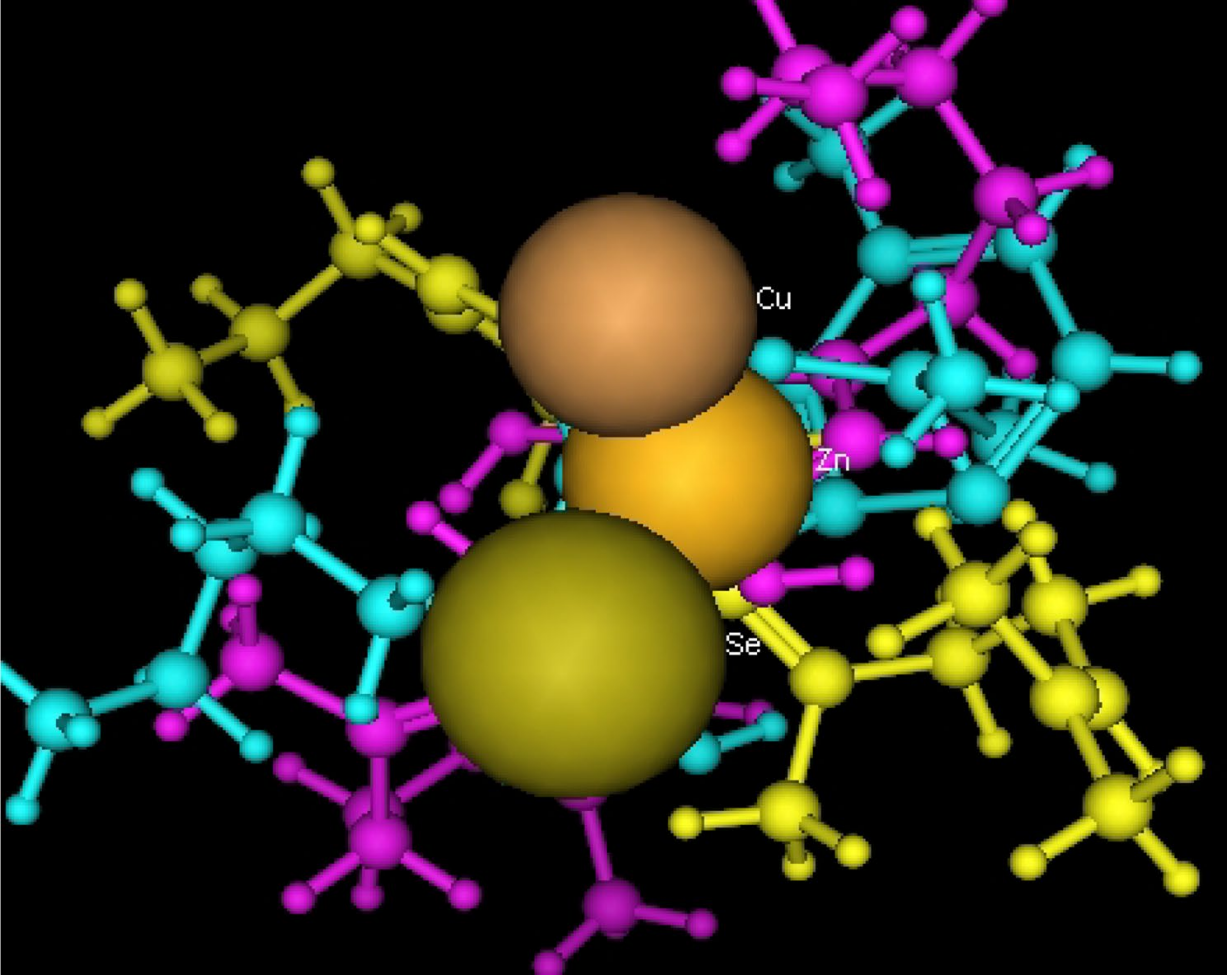
Copper-Zinc-Selenium (Cu-Zn-Se) nanocomposite with active plant metabolites

**Fig. 17:**
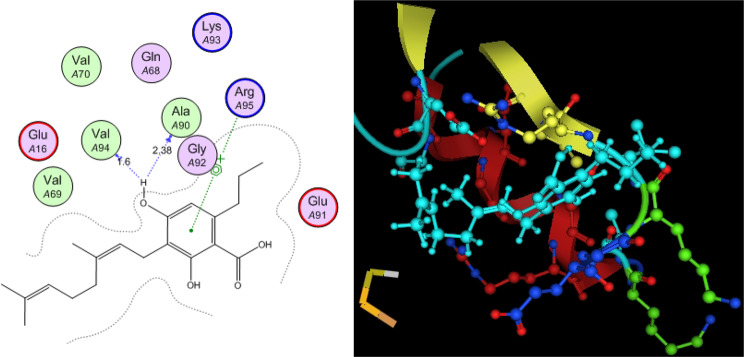
2D & 3D docked pose of standard drug Ascorbic acid

**Fig. 18:**
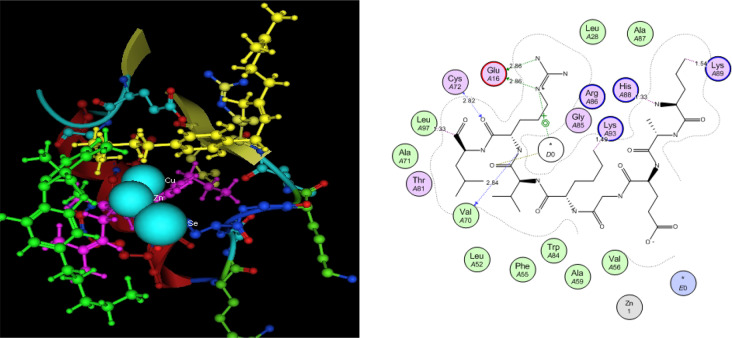
3D & 2D docked view of Cu-Zn-Se nanocomposite with *Cannabis sativa*, green dotted line shows H-binding, green sphere show hydrophobic interactions

Utilizing in silico screening for identifying inhibitory molecules presents a promising approach to addressing chronic and degenerative diseases by discovering selective, non-toxic inhibitors. This strategy plays a crucial role in drug discovery, particularly in conditions linked to oxidative stress. A fundamental aspect of drug design and development involves evaluating the binding affinities and interaction efficiency of potential compounds with specific biological targets (Maraldi et al., 2013). This study aims to identify potential natural inhibitors through molecular docking, an efficient and precise method that enables rapid screening of extensive compound libraries while providing detailed interaction profiles at the atomic level. The antioxidant potential of a Copper-Zinc-Selenium (Cu-Zn-Se) nanocomposite incorporating cannabinoids such as CBD, THC, and cannabinol was analyzed against the target ligand 3MNG using molecular docking (Figs. 11, 12 and 13). The structural composition of the Cu-Zn-Se nanocomposite combined with bioactive plant metabolites is illustrated in Figs. 14, 15 and 16. Additionally, docking results for ascorbic acid, a widely known antioxidant, with 3MNG are shown in Fig. 17, serving as a reference for comparison. Docking analysis revealed that ascorbic acid interacts with 3MNG through hydrogen bonding and hydrophobic interactions, which are essential for its antioxidant properties. These interactions were visualized using green dotted lines for hydrogen bonds and green spheres for hydrophobic interactions in the docked poses (Joozdani et al., 2022). Similarly, docking results for the Cu-Zn-Se nanocomposite with 3MNG, as shown in Fig. 18, demonstrated significant hydrophobic and hydrogen bonding interactions. The active site of 3MNG exhibited strong interactions with key amino acid residues, including serine (Ser), asparagine (Asn), valine (Val), and phenylalanine (Phe), contributing to the stability and effectiveness of the nanocomposite in antioxidant activity (Pavlovic et al. 2019). The presence of CBD, THC, and cannabinol in the nanocomposite further enhanced its antioxidant properties, as cannabinoids are well-documented for their ability to scavenge free radicals and reduce oxidative stress (Pagano et al. 2023). Docking results supported this, showing strong binding affinities and stable interactions between the nanocomposite and 3MNG. The binding interactions and energy values reinforced the antioxidant potential of the nanocomposite (Kader et al. 2024). The docking interactions were visualized in 2D and 3D docked poses, highlighting specific binding sites and interaction types. Furthermore, a strong correlation was observed between the theoretical docking results and experimental antioxidant assays, confirming the reliability of computational predictions. These findings support the potential application of nanocomposites enriched with bioactive plant metabolites, such as cannabinoids, for the development of innovative antioxidant therapies (Fig. 18). Future studies should focus on further validating the antioxidant and therapeutic potential of the Cu-Zn-Se nanocomposite enriched with cannabinoids through in vitro and in vivo experiments. Investigating its mechanistic pathways in oxidative stress-related diseases, could provide valuable insights into its pharmacological applications. Advanced computational techniques, such as molecular dynamics simulations, could further refine the understanding of its binding interactions with key biological targets (Naqvi et al. 2018). Future research should also consider nanoparticle functionalization strategies to enhance its stability, bioavailability, and selective targeting of diseased cells. Ultimately, integrating this nanocomposite into nanomedicine-based therapeutic formulations could open new avenues for the development of next-generation antioxidant and anticancer treatments.

## Conclusion

The present study successfully demonstrates the synthesis, characterization, and evaluation of the antioxidant activity of the Cu-Zn-Se nanocomposite using *Cannabis sativa* floral biomass extract. The GC-MS analysis effectively identified the presence of key cannabinoids (THC, CBD, and CBN) and terpenes in the cannabis extract. These compounds contribute to the overall effects and therapeutic potential of the cannabis sample. Various techniques such as UV-Vis spectroscopy, FTIR, and SEM were employed to confirm the formation and composition of the nanocomposite. The results from these analyses indicated the successful biosynthesis of the Cu-Zn-Se nanocomposite with spherical particles of an average size of 60 nm. Antioxidant properties of the Cu-Zn-Se nanocomposite were evaluated by various assays, such as DPPH free radical scavenging, hydrogen peroxide scavenging, ferric reducing antioxidant power, and phosphomolybdate assays. The nanocomposite was found to possess useful antioxidant properties, which were effective across all assays and comparable to those of ascorbic acid, an established antioxidant. Remarkably, the antioxidant activity of the Cu-Zn-Se nanocomposite was found to increase with concentration, making it possible for future work of related significance to utilize the substance in various antioxidant-based applications. The results of molecular docking studies further confirmed the experimental results indicating that the Cu-Zn-Se nanocomposite fitting stably in the binding pocket of the antioxidant target protein thereby strengthening its probable antioxidant activity. These results signify the immense bioactive potential of the green synthesized Cu-Zn-Se nanocomposite using *Cannabis sativa* extract, which can find applications in the pharmaceutical and industrial fields. While this investigation offers encouraging insights into the antioxidant capabilities of the Cannabis sativa-derived Cu-Zn-Se nanocomposite, comprehensive structural characterization through methods like X-ray diffraction (XRD) and high-resolution transmission electron microscopy (HRTEM) along with extensive in vivo bioactivity assessments, remains essential for a complete understanding of its internal architecture and therapeutic potential.

## Data Availability

No datasets were generated or analysed during the current study.
